# Prognostic significance of p21, p27 and survivin protein expression in patients with oral squamous cell carcinoma

**DOI:** 10.3892/ol.2013.1381

**Published:** 2013-06-07

**Authors:** MINGBIN ZHANG, JIANG LI, LIZHEN WANG, ZHEN TIAN, PING ZHANG, QIN XU, CHENPING ZHANG, FENGCAI WEI, WANTAO CHEN

**Affiliations:** 1 School Of Stomatology, Shandong University, Jinan, Shandong 250012;; 2 Department of Stomatology, Tai’an City Central Hospital, Tai’an, Shandong 271000;; 3 Shanghai Key Laboratory of Stomatology, Ninth People’s Hospital, Shanghai Jiao Tong University School of Medicine, Shanghai 200011 P.R. China

**Keywords:** p21, p27, survivin, oral squamous cell carcinoma, biomarker, prognosis

## Abstract

Oral squamous cell carcinoma (OSCC) accounts for >80% of head and neck malignancies. p21, p27 and survivin proteins are abnormally expressed in OSCC and have been previously reported to correlate with cell proliferation and apoptosis. However, the prognostic significance of p21, p27 and survivin remains controversial. The aim of the present study was to investigate the association of clinical parameters and prognosis with the levels of p21, p27 and survivin expression in patients with OSCC. The levels of the three biomarkers were evaluated by immunohistochemical staining in specimens from 110 patients with OSCC and each section was scored according to the percentage of positive tumor cells and staining intensity. Log-rank test and Cox proportional hazards regression were performed to assess the correlation between biomarkers and clinical events. The association between the immunoexpression of p21, p27 and survivin and clinical pathological variables were analyzed by the χ^2^ test and a non-parametric analysis. The expression of p21 in patients with OSCC was found to correlate with the expression of p27 and survivin. The results of the current study revealed that the five-year survival rate was significantly lower in patients with high p21 expression. In addition, the expression of p27 also showed a negative correlation with the five-year survival rate of OSCC, but to a lesser extent. By contrast, the expression of survivin was not a prognostic factor for OSCC. A Kaplan-Meier analysis and Cox proportional hazards model showed that lymph node metastasis and p21 expression were independent prognostic factors of OSCC.

## Introduction

Oral squamous cell carcinoma (OSCC) is the most prevalent pathological oral cancer, accounting for >80% of head and neck malignancies (1). The carcinogenesis of OSCC is a multistage process involving the activation of oncogenes and the inactivation of tumor suppressor genes, with a cellular imbalance between cell death and growth. Regulators of apoptosis and the cell cycle may be important for the cellular balance of cell death and growth in cancer.

p21, p27 and survivin proteins have been identified to be abnormally expressed in the majority of human forms of cancer, including oral carcinoma. These expression levels usually correlate with cell proliferation and apoptosis, which contribute to the molecular carcinogenesis of cancer (2–5). The overexpression of p21 and p27 enhances the binding to cyclin-CDK complexes, which inhibits cell proliferation (6). Previous studies have shown that, in conjunction with survivin, p21 and p27 may also inhibit cell apoptosis (7,8). The correlation between patient prognosis and biomarker expression has received considerable interest, however, the clinical implications and prognostic value of this correlation remain controversial due to the complex mechanisms of carcinogenesis and limitations with regard to small sample sizes and short follow-up periods (9–13). In the current study, the long-term follow-up of p21, p27 and survivin immunoexpression was extensively monitored in 110 patients with OSCC to examine the association with prognosis.

## Material and methods

### Study participants

p21, p27 and survivin expression levels were identified in formalin-fixed and paraffin-embedded specimens from 110 OSCC patients admitted to the Department of Oral and Maxillofacial Surgery (The Ninth People’s Hospital, Shanghai Jiao Tong University School of Medicine, Shanghai, China) between 1989 and 1993. Oral mucosa samples from 20 healthy participants were included as controls. All individuals were treated by standard radical surgery with negative margins, and patients who were classified with T3, T4 or lymph node metastasis, according to the International Union Against Cancer TNM classification (14), were treated with 50–65 Gy radiotherapy post-operatively. Patients were followed up for >5 years. The mean age and range of the patients was 58 and 37–78 years-old, respectively. Gender, pathological grade and clinical stage are shown in Table I. Pathological grade was independently evaluated by three experienced pathologists. This study was approved by the ethics committee of The Ninth People’s Hospital, Shanghai Jiao Tong University School of Medicine, Shanghai, China. Written informed consent was obtained from the patient’s family.

### Immunohistochemical staining

Immunostaining for p21 (ZM-0206), p27 (ZM-0340) and survivin (ZA-O530) was performed using reagents from Beijing Zhongshan Golden Bridge Biotechnology Co., Ltd. (Beijing, China) according to the manufacturer’s instructions. Sections were dewaxed in xylene and rehydrated in graded alcohol prior to pretreatment with 0.3% hydrogen peroxide in phosphate-buffered saline (PBS) for 15 min to block endogenous peroxidase. Following this, a further 3 PBS washes were performed. The dewaxed sections were heated in a microwave oven in 10 mM citric acid buffer (pH 6.0) for 15 min and gradually cooled down to room temperature. The sections were incubated with appropriate antibodies (mouse anti-human p21, p27 and rabbit anti-human survivin) overnight in a humidified chamber at 4°C. The sections were then washed 3 times with PBS and incubated for 1 h at room temperature in a humidified chamber with a corresponding second antibody (goat anti-mouse and goat anti-rabbit), followed by being washed with PBS. Next, the sections were washed with a developing solution containing 0.06% diaminobenzidine and 0.1% hydrogen peroxide and then counterstained with hematoxylin and mounted. The sections without primary antibodies or with non-immunized rabbit serum for p21, p27 and survivin were included as the negative controls.

The sections were examined microscopically by three pathologists and scored according to the fraction of stained tumor cells and the staining intensity (Table II). The mean of each protein expression level was used as a cut-off value to define high and low expression (15), for example, p21 expression was high if the p21 score was >2.7 and low if ≤2.7. Similarly, the p27 and survivin cut-off values were 2.8 and 2.0, respectively.

### Statistical analysis

The results were analysed using SAS package version 9.2 (SAS Institute Inc., Cary, NC, USA). Prognostic factors were evaluated by univariable and multivariable analyses in the Cox proportional hazards model, and the correlation between all the parameters was analyzed using Pearson’s correlation. P<0.05 was considered to indicate a statistically significant difference.

## Results

### Expression of p21 and p27 in OSCC cells

**Figure 1.**
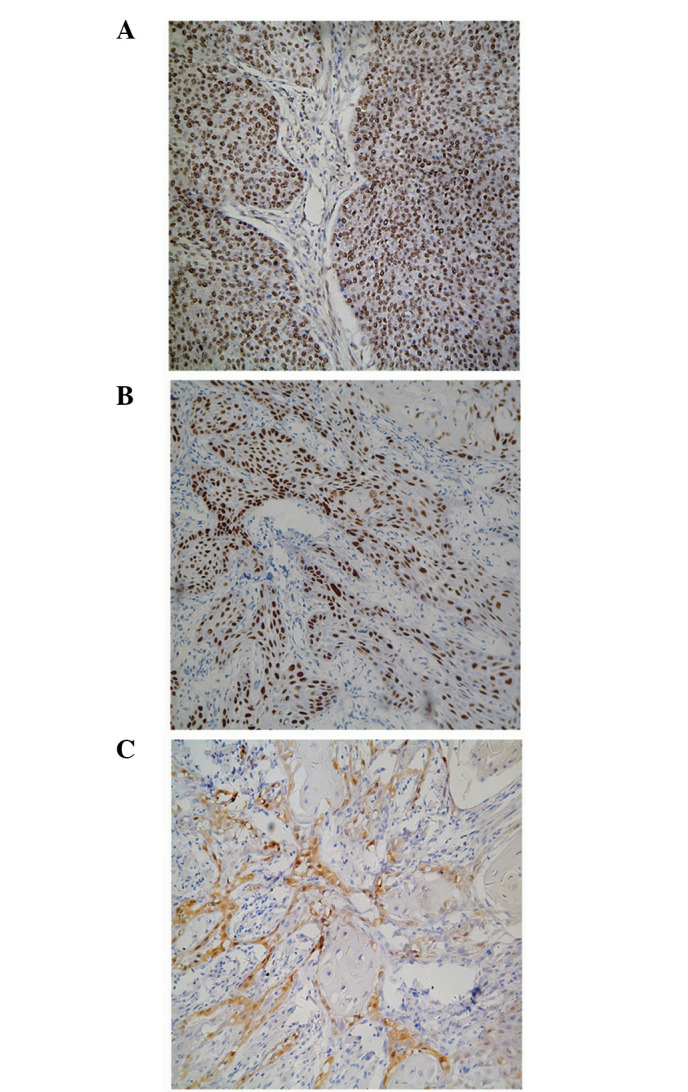
Immunohistochemical staining of (A) p21 and (B) p27 in OSCC, observed in the nuclei. (C) Staining of survivin located in the cytoplasm. Magnification, ×100. OSCC, oral squamous cell carcinoma.

**Figure 2.**
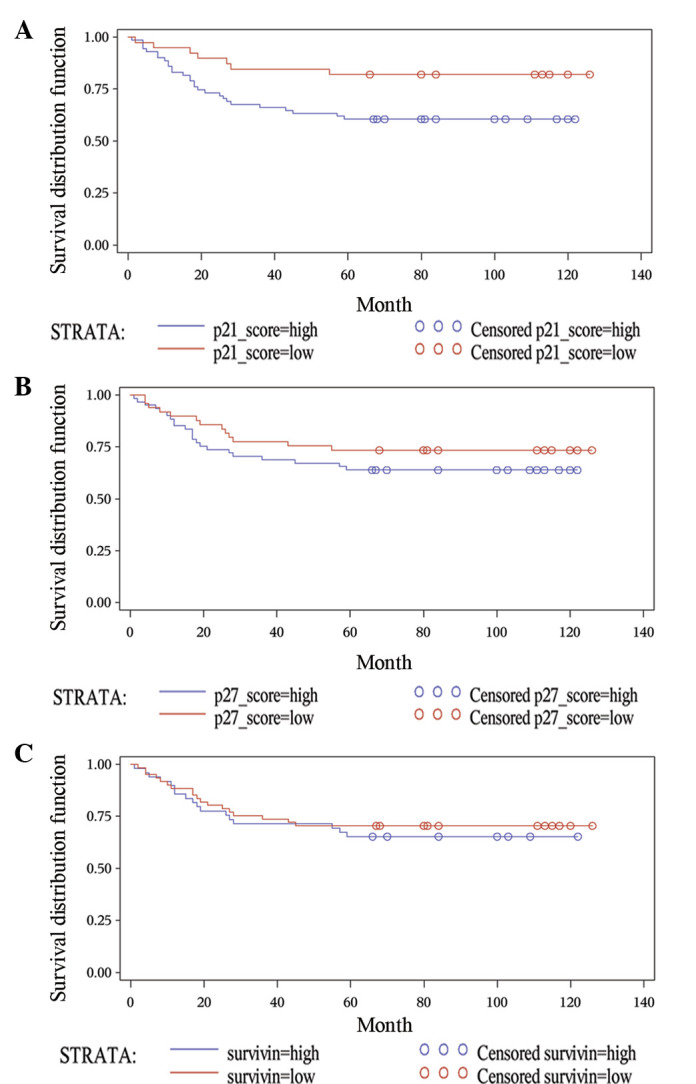
Kaplan-Meier plots for high vs. low expression of (A) p21 (B) p27 and (C) survivin in patients with OSCC. OSCC, oral squamous cell carcinoma.

The majority of p21 expression was localized in the nucleus of the OSCC cells and was identified by dark staining (Fig. 1A). The percentage of cells with positive p21 expression was 64.55% with a mean score of 2.7±0.8. A log-rank test showed that the five-year survival rate of patients with elevated p21 expression levels was significantly lower when compared with that of patients with low p21 expression levels (60.56±5.80 vs. 82.05±6.15%; P=0.0222; Fig. 2A). Similarly, the majority of p27 expression was localized in the nucleus of the OSCC cells, however, the staining intensity was moderate (Fig. 1B). The percentage of cells with positive p27 expression was 55.46%, with a mean score of 2.8±0.9. Patients with an elevated p27 level exhibited a lower five-year survival rate when compared with that of patients with lower p27 expression levels (63.93±6.15 vs. 73.47±6.31%; P=0.2777), however, this was not statistically significant (Fig. 2B).

### Expression of survivin in OSCC cells

By contrast, the majority of survivin was expressed in the cytoplasm of the OSCC cells, with negligible detection in the nucleus (Fig. 1C). The percentage of cells with positive survivin expression was 64.55%, with a mean score of 2.0±1.0. No significant difference was identified between the five-year survival rate of patients with an elevated level of survivin expression when compared with that of patients with lower survivin expression levels (65.31±6.80 vs. 70.49±5.84%; P=0.5843; Fig. 2C).

### Correlation between p21, p27 and survivin and various parameters, and the univariate analysis results

The Pearson’s correlation analysis (Table III) revealed that p21 expression significantly correlated with p27 (r=0.210; P=0.026) and survivin (r=0.292; P=0.002) expression. In addition, there was a significant correlation between lymph node metastasis and tumor size (r=0.229; P=0.016). However, statistical correlations were not found between the immunoexpression of p21, p27 and survivin and age, gender, tumor size, lymph node metastasis or pathological grade (P>0.05).

The univariate analysis indicated that among the observed variables, only p21 expression and the status of lymph node metastasis were associated with the prognosis of patients with OSCC (Table IV).

## Discussion

p21 and p27 are members of the Cip/Kip gene family that inhibit cell cycle progression by binding cyclin/Cdk complexes, thus functioning as regulators of cell cycle progression at the G_1_ stage (16,17). In addition, the Cip/Kip family has been identified to activate the cyclin D/Cdk4 complex (18–22). p21 is capable of stabilizing the Cdk4-cyclin D interaction and promoting the formation of active complexes in a dosage-dependent manner (19). p21 may also regulate apoptosis (23,24) through interactions with p27 and survivin (7,8). p21 functions as an inhibitor of apoptosis in a number of systems, which may counteract its tumor-suppressive function as a growth inhibitor (25). p21 has been reported to regulate apoptosis via p53-dependent and -independent pathways (26–28).

In the present study, the percentage of p21 and p27-positive cells was ∼64.55 and 55.46%, respectively. The results showed that the expression of p21 (P=0.0222) and p27 (P=0.2777) negatively correlated with prognosis, however, this was not statistically significant for p27. The present study demonstrated that the five-year survival rate of patients with a p21 expression score of >2.7 was significantly lower when compared with that of patients with a score of ≤2.7, inconsistent with the majority of previous studies in OSCC (10,11) and other types of cancer (29–34). However, certain other controversial results have also been reported (35–37).

Survivin is a member of the inhibitor of apoptosis protein family, and specific expression levels of survivin have been identified in embryogenesis and tumor cells (38–40). Survivin has been shown to be involved in cell division, anti-apoptosis and cell cycle control (40–44), and it has been hypothesized that survivin interacts with p21 to regulate cell apoptosis (7,8). The survivin-p21 axis is important for the proliferation of normal hematopoietic cells and in the regulation of apoptosis through the p21WAF1/Cip1-dependent pathway (45). In the current study, the percentage of cells with survivin expression was ∼64.55%, similar to results from previous studies (46). However, inconsistent with these previous results, survivin was not found to be an independent prognostic factor of OSCC (47,48).

The expression of p53 and Bcl-2 was also examined in the samples (data not shown) and, notably, p21 expression was shown to be correlated with p27, survivin and Bcl-2, but not p53, indicating that p21 may function in a p53-independent manner in OSCC. The coexpression patterns among p21, p27, Bcl-2 and survivin demonstrated that p21 plays a predominant role in inhibiting apoptosis, likely through interactions with p27 and survivin (7,8). In addition, p21-expressing cells may produce antiapoptotic proteins that affect the survival of adjacent cells through a paracrine effect (49). It has also been hypothesized that by inhibiting apoptosis in OSCC, p21 may enable tumor cells to accumulate for cell proliferation and may also present resistance to therapy by inhibiting treatment-related apoptosis, resulting in a reduced five-year survival rate (50). This may explain the negative correlation between the overexpression of p21 and prognosis.

In conclusion, the current study reveals that the prognosis of OSCC may be affected by a number of clinical pathological factors and biomarkers, among which, p21 plays an important role by inhibiting cell apoptosis or resistance to therapy. To improve overall survival rates, patients with a high p21 expression level must be administered intensive combined therapy and provided with follow-ups at an increased frequency.

## Figures and Tables

**Table I.** Baseline characteristics of OSCC patients.

Clinical observations	n	Percentage
Gender		
Male	59	53.64
Female	51	46.36
Age, years		
≤60	75	68.18
>60	35	31.82
Tumor size		
T1	14	12.73
T2	53	48.18
T3	16	14.55
T4	27	24.54
Lymph node metastasis		
N0	82	74.55
N1	25	22.73
N2	3	2.72
Pathological grading		
I–II	94	85.45
III	16	14.55

The tumor size is according to the TNM classification grade. OSCC, oral squamous cell carcinoma.

**Table II.** Classification standard for immunohistochemical staining of p21, p27 and survivin, evaluation and expression score calculation.

Standard	Score
Percentage	
0	0
5	1
≤25	2
≤50	3
>50	4
Intensity	
Negative	0
Weak	1
Moderate	2
Intense	3

**Table III.** Pearson’s correlation between parameters observed in patients with OSCC.

Parameter	Gender	Age	Pathological grade	Tumor size	Node metastasis	p21	p27	Survivin
Gender	1.000	−0.002	−0.030	0.182	−0.023	−0.022	0.128	0.190
Age, years	−0.002	1.000	−0.056	0.051	0.049	−0.153	−0.104	−0.182
Pathological grade	−0.030	−0.056	1.000	0.100	−0.082	0.104	0.106	−0.039
Tumor size	0.182	0.051	0.100	1.000	0.229	−0.027	0.050	−0.011
Node metastasis	−0.023	0.049	−0.082	0.229	1.000	−0.120	0.006	0.044
p21	−0.022	−0.153	0.104	−0.027	−0.120	1.000	0.210	0.292
p27	0.128	−0.104	0.106	0.050	0.006	0.210	1.000	0.212
Survivin	0.190	−0.182	−0.039	−0.011	0.044	0.292	0.212	1.000

Tumor size is according to the TNM classification grade. OSCC, oral squamous cell carcinoma.

**Table IV.** Univariate analysis of prognostic factors for survival using the Cox proportional hazards model.

Clinicopathological parameters	5-year survival rate, %	χ^2^	P-value
Gender			
Male	66.10±6.16	0.2087	0.6478
Female	70.59±6.38		
Age, years			
≤60	64.00±5.54	1.8642	0.1721
>60	77.14±7.10		
Tumor size			
T1–T2	73.13±5.42	1.8661	0.1713
T3–T4	60.47±7.46		
Node metastasis			
Positive	75.61±4.74	10.8832	0.0011
Negative	46.43±9.42		
Pathology grading			
I–II	70.21±4.72	1.2445	0.2646
III	56.25±12.40		
p21 expression			
≤2.7	82.05±6.15	5.2307	0.0222
>2.7	60.56±5.80		
p27 expression			
≤2.8	73.47±6.31	1.1783	0.2777
>2.8	63.93±6.15		
Survivin expression			
≤2.0	70.49±5.84	0.2993	0.5843
>2.0	65.31±6.80		

Tumor size is according to the TNM classification grade.
